# Electricity and catholyte production from ceramic MFCs treating urine

**DOI:** 10.1016/j.ijhydene.2016.09.163

**Published:** 2017-01-19

**Authors:** Irene Merino Jimenez, John Greenman, Ioannis Ieropoulos

**Affiliations:** a Bristol BioEnergy Centre, Bristol Robotics Laboratory, University of the West of England, BS16 1QY, UK; b Biological, Biomedical and Analytical Sciences, University of the West of England, BS16 1QY, UK

**Keywords:** Microbial Fuel Cell (MFC), Ceramic membrane, Catholyte production, Electroosmotic drag, Urine

## Abstract

The use of ceramics as low cost membrane materials for Microbial Fuel Cells (MFCs) has gained increasing interest, due to improved performance levels in terms of power and catholyte production. The catholyte production in ceramic MFCs can be attributed to a combination of water or hydrogen peroxide formation from the oxygen reduction reaction in the cathode, water diffusion and electroosmotic drag through the ion exchange membrane. This study aims to evaluate, for the first time, the effect of ceramic wall/membrane thickness, in terms of power, as well as catholyte production from MFCs using urine as a feedstock. Cylindrical MFCs were assembled with fine fire clay of different thicknesses (2.5, 5 and 10 mm) as structural and membrane materials. The power generated increased when the membrane thickness decreased, reaching 2.1 ± 0.19 mW per single MFC (2.5 mm), which was 50% higher than that from the MFCs with the thickest membrane (10 mm). The amount of catholyte collected also decreased with the wall thickness, whereas the pH increased. Evidence shows that the catholyte composition varies with the wall thickness of the ceramic membrane. The possibility of producing different quality of catholyte from urine opens a new field of study in water reuse and resource recovery for practical implementation.

## Highlights

• The effect of the membrane thickness is evaluated for clay MFCs treating urine.
• Power performance increased for the thinner MFCs, reaching 2.1 ± 0.19 mW at 2.5 mm.
• Power increased by 50% reducing the membrane thickness from 10 to 2.5 mm.
• The ceramic thickness affects the ORR, catholyte production rate and properties.
• Thicker MFCs generate higher alkali catholyte for recycling and nutrients recovery.

## Introduction

Microbial Fuel Cells (MFCs) present an attractive approach to renewable energy for electricity production from waste [1]. The research on electricity production from organic compounds has been investigated since the beginning of the 20th century [2]. Since then, the generation of voltage and current from several types of organic compounds including glucose, acetate and waste water, has been thoroughly investigated. However, the use of urine as a feedstock for MFCs offers a more recent approach, being reported for the first time in 2012 [3]. Urine is an abundant fuel with a daily production of 17.4 billion litres, based on a world population of 6.97 billion and considering that an adult produces an average of 2.5 L in a day [3]. In MFCs, the chemical energy in urine can be directly converted into electricity using microorganisms as biocatalysts. An MFC normally comprises two chambers, the anodic and the cathodic, that are separated by an ion exchange membrane, which can also act as support material, being sandwiched between the anode and cathode electrodes. The anodic chamber contains the microorganisms that break down the organic matter in urine into smaller molecules, whilst releasing electrons that travel through the wire to the cathode electrode, and protons that pass through the ion exchange membrane to the cathodic chamber. In the cathodic chamber, the oxygen reduction reaction (ORR) takes place by consuming oxygen from air to generate water or hydrogen peroxide, depending on the catalyst used. The ORR is one of the major limiting factors in fuel cells, especially in MFCs which operate at neutral pH and room temperature [4].

The efficiency of MFCs can also be limited by several other factors, such as microbial community on biofilm anode, internal resistance and many others [4]. The use of an electrochemically active catalyst on the cathode electrode, which can improve the ORR kinetics, and the use of membrane materials, with high ionic conductivity and low ohmic resistance, could improve the MFC power performance. An electrochemically efficient catalyst, such as Pt or Pt alloys, would promote the ORR through a 4e^−^ pathway, which in an alkaline medium would take place according to reaction (1):

(1) $$O_{2}+2H_{2}O+4e^{-}\to 4OH^{-},\;E^{o}=0.401\;Vvs.SHE$$

This reaction can also take place in two steps, following a hydrogen peroxide formation and oxidation pathway:

(2) $$O_{2}+H_{2}O+2e^{-}\to OOH^{-}+OH^{-},\;E^{o}=-0.065\;Vvs.SHE$$

(3) $$HO_{2}^{-}+H_{2}O+2e^{-}\to 3OH^{-},\;E^{o}=0.867\;Vvs.SHE$$

However, when using non-noble metal catalysts, the reduction of hydrogen peroxide in alkaline media usually follows a 2e^−^ pathway and hydrogen peroxide degradation. Therefore, the ORR could involve either the 2 or 4e^−^ pathway depending on the catalyst used. However, the majority of the highly effective catalyst materials are unaffordable for a commercially viable technology. In terms of catalysts for the ORR, there has been an extensive line of work for low cost cathode electrodes for MFCs, which has given rise to activated carbon based materials since it offers performance stability at lower cost [10], [11]. In an effort to find low-cost effective materials to reduce the manufacturing cost of MFCs, ceramics have been reported as a good candidate for successfully substituting expensive commercially available ion-exchange membranes [5], [6], [7], [8]. The interest in using ceramic as low cost membrane materials for MFCs is receiving increasing attention also because of the improved performance in terms of power and catholyte production [9]. However, the optimisation of such materials, including composition, porosity and wall thickness, needs to be studied further. Recent studies demonstrated simultaneous electricity generation from MFCs and catholyte accumulation in the cathode compartment without the need for external power [12]. This accumulation of catholyte, in the initially empty cathode chamber, was attributed to the combination of the following factors: (i) the water produced as a result of the ORR in the cathode electrode; (ii) the passive water diffusion across the membrane; and (iii) when the MFC was under load, the electroosmotic drag of water molecules together with the cations that migrate from the anode to the cathode [13]. The catholyte generation, in this case, has several advantages including the self-hydration of the ionic exchange membrane, the hydration of the cathode electrode, and the possibility to extract such solution for other applications, such as fertiliser enrichment. Although the hydration of the membrane and the catalyst layer benefits the ion transfer and the electrode kinetics [13], [14], water accumulation at the cathode side might also lead to an increase in membrane resistance and consequent decrease in fuel cell performance [15]. Therefore, a rigorous analysis of the effect of the catholyte accumulation in the MFC power output should be performed.

In this study, the effect of wall thickness of fine fire clay (FFC) ceramic membranes for MFCs treating urine was evaluated, in terms of power generation and catholyte accumulation. For this purpose cylindrical MFCs were assembled with ceramics of three different thicknesses (2.5, 5 and 10 mm), as structural and membrane materials. The catholyte produced from the MFCs was analysed in terms of volume, pH and mineral composition. The correlation between the catholyte produced, its composition and the effect of the membrane thickness was evaluated for both catholyte production and power output.

## Methods

### MFC assembly

**Fig. 1:**
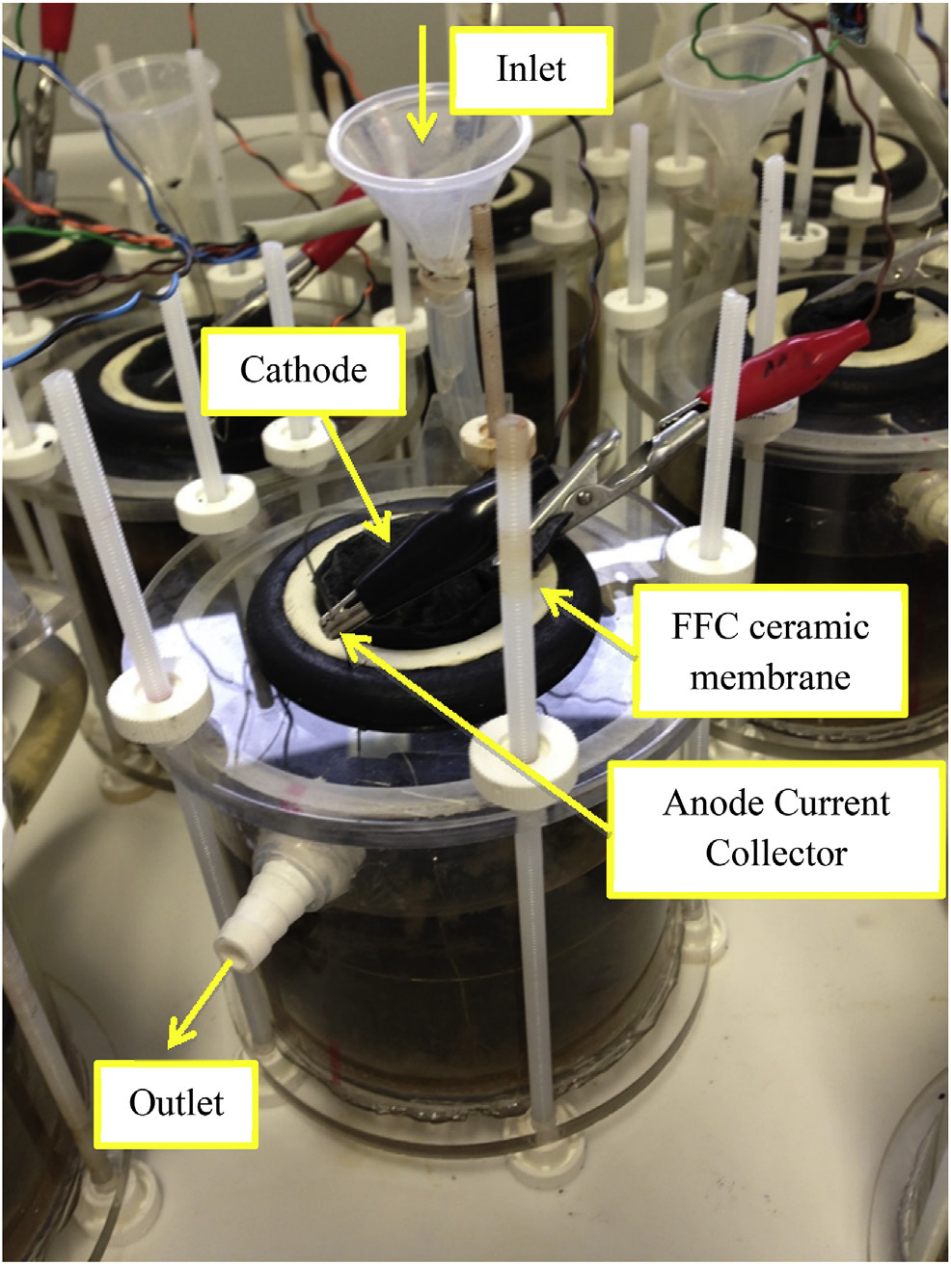
Photo of the MFC setup.

Cylindrical ceramic MFCs were assembled using fine fire clay cylinders (ROCA, Spain) with three different wall thicknesses 2.5, 5 and 10 mm as membranes. The ceramics were tested in triplicates and control MFCs of each thickness, were left at open circuit throughout the whole experiment. All the cylinders had 84 mm height and an external diameter of 48 mm. The anode electrode was constructed from 90 × 27 cm^2^ untreated carbon veil with a density of 30 g/m^2^ (PRF Composites, Dorset, UK), which was folded and wrapped around the external surface of the ceramic cylinder. Stainless steel wire (0.5 mm, Scientific Wire Company) was threaded through the electrodes and used as a current collector. Once the anode electrode was wrapped around the ceramic membrane, the cylindrical MFC was housed in a separate acrylic cylinder, forming the anode chamber, with a top and bottom acrylic lids, bolted together, as shown in Fig. 1. The internal volume of the anode chamber for each MFC was 200 mL. The inlet was introduced from the bottom and the outlet discharged from the top of the container to optimise the distribution of fresh urine through the anodic chamber. The cathode electrode consisted of a gas diffusion electrode with carbon veil as the support material and a microporous layer (MPL), which was prepared with a mixture of activated carbon (GBaldwin&Co, 80 g/140 mL solution), polytetrafluoroethylene (PTFE) (60% wt. Sigma–Aldrich) and distilled water, as previously described [16]. The cathode electrodes were cut with a surface area of 65 cm^2^ and placed inside the ceramic cylinders. A stainless steel crocodile clip was connected to the cathode electrodes as a current collector and acrylic rings were placed inside the ceramic cylinder to improve contact between the cathode electrode and the ceramic membrane.

The images of the ceramic structure were captured using a Philips XL30 scanning electron microscope (SEM). Energy dispersive X-ray (EDX) analysis was also performed (Philips XL30 SEM) and was used to determine elements present in the ceramic material. For improved visualisation of the sample in the microscope, the ceramic samples were PVD gold coated at 10 milliamps for 5 min using an Emscope SC500 sputter coating unit.

### Inoculation process

The MFCs were inoculated with a mixture of 50% activated sewage sludge supplied from the Wessex Water Scientific Laboratory (Saltford, UK) and 50% fresh urine, which was donated by healthy individuals aged between 18 and 70 years old, with a normal diet and no known medical conditions. During the first day, the MFCs were left open circuit for the first two hours from inoculation, following which a 2 kΩ external resistance was connected to each cell. The inoculum was replaced on a daily basis for three days. After the third day, a continuous feeding of only fresh urine was established with each MFC being fed directly from the inlet reservoir using a 16-channel peristaltic pump (205 U, Watson Marlow, Falmouth, UK). The flow rate was set to 9 mL h^−1^ giving a hydraulic retention time (HRT) of 22 h, which was retained throughout the experiment. After the first polarisation experiment, the external resistance was changed to 100 Ω, a value at which maximum power was generated, which remained constant throughout the experiment. All experiments were performed at room temperature 22 ± 2 °C.

### Data collection

Each MFC was individually monitored by recording the cell voltage in volts (V) against time by using an Agilent data logging (KEYSIGHT, 34972A LXI data acquisition/Switch) unit. The current and power produced from the MFCs were calculated using Ohm's law (*I* = *V/R*), and *P* = *I* × *V*, respectively, where the external resistance applied was of a known value. The internal resistance (*R*_*INT*_) was calculated using Eq. (4) [17]:

(4) $$R_{INT}=\left(\frac{OCV}{I_{L}}\right)-R_{EXT}$$

where *OCV* is the open circuit voltage of the MFC, *I*_*L*_ is the current under a given load and *R*_*EXT*_ is the given load.

### Polarisation

Polarisation experiments were performed using a DR07 decade variable resistor box (ELC, France), within the range of 30 KΩ and 3.74 Ω, applying each resistance for 5 min. During polarisation, the cathode redox voltage was also monitored with a separate Ag/AgCl reference electrode (1 M KCl, Sigma–Aldrich). The anode voltage was calculated from the overall cell voltage and the cathode voltage that were measured during the polarisation, using Eq. (5) [18]:

(5) $$V_{MFC}=\left(V_{Cathode}-V_{Anode}\right)-\sum IR$$

where ∑IR corresponds to all the voltage drop values due to the combination of the ohmic losses, the electrolyte losses and those from the membrane internal resistance. Therefore, all the aforementioned losses form part of the *V*_*Anode*_, in the plotted data (Fig. 4). The electrode voltage versus Ag/AgCl were converted with reference to SHE (standard hydrogen electrode) for a better comparison with the literature, by adding 230 mV to each electrode voltage value.

### Catholyte collection and analysis

The catholyte generated was collected every 7 days using a sterile syringe. The pH and conductivity were measured using a Hanna 8424 pH meter (Hanna, UK) and a 470 Jenway conductivity meter (Camlab, UK), respectively. Dry weight of precipitated salts was determined by drying 1 mL of catholyte over 48 h and weighing the dry mass.

## Results and discussion

**Fig. 2:**
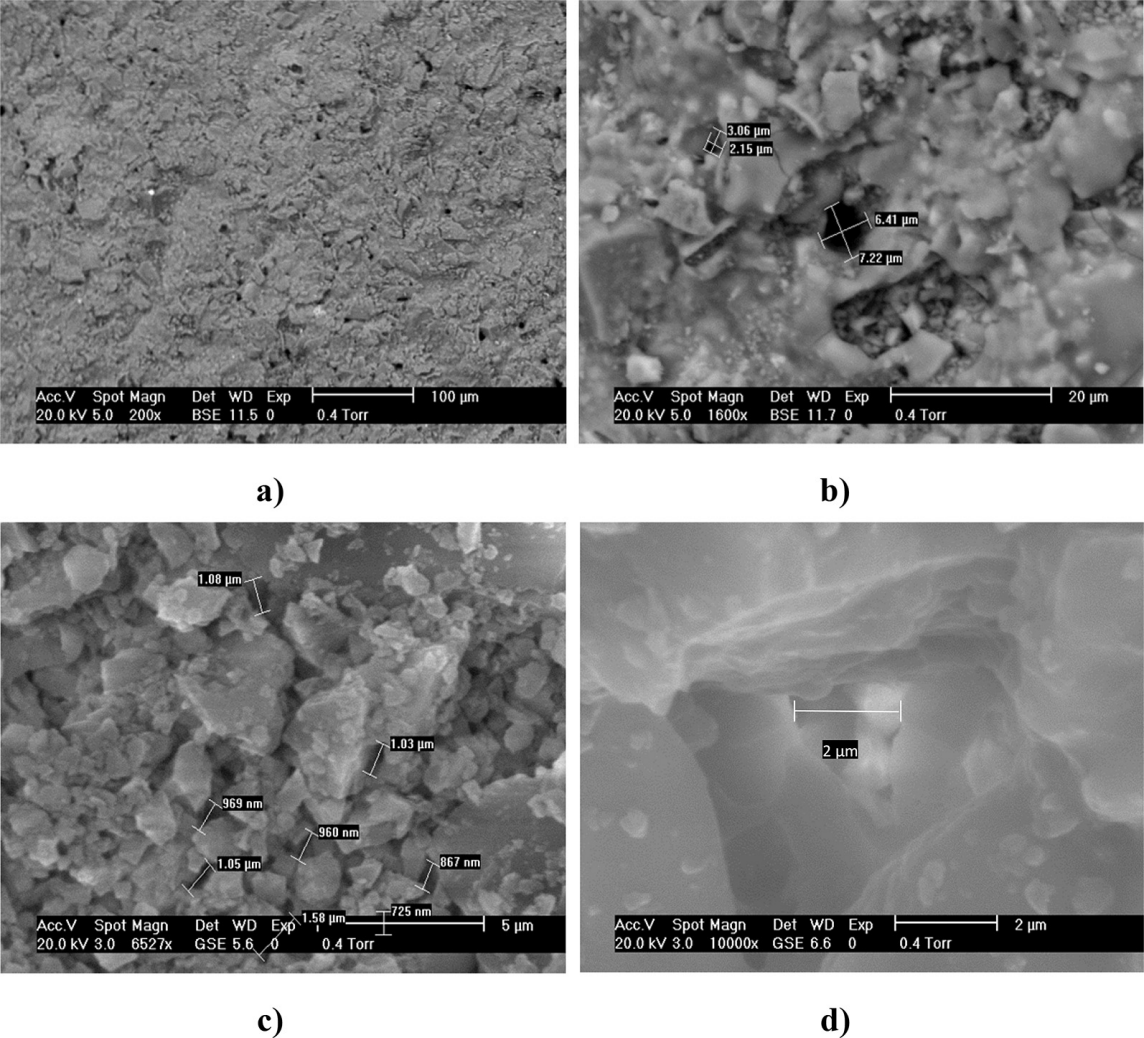
ESEM images of the fine fire clay of 2.5 mm; (a) and (b) are from the internal area, whereas (c) and (d) are side views of the ceramic cylinder at different magnifications.

Fig. 2 shows the SEM images of the fine fire clay membrane of 2.5 mm thickness. As can be seen in Fig. 2a, the fine fire clay has a porous structure with a non-uniform pore size. Fig. 2b shows the differences in pore size, with one pore having a diameter of more than 6 μm, whereas the second is of 2.5 μm diameter. Fig. 2c shows a more homogeneous pore size, corresponding to the middle part of the cylinder thickness, with an average pore size of approximately 1 μm. This suggests that the pore size was bigger on the surface of the material and it became smaller and more uniform, towards the centre. Therefore, the ceramic had different pore sizes on the surface that varies from 7.2 μm to 3 μm and smaller, reaching an average of 1 μm in the central part of the ceramic wall. This can be seen in Fig. 2d, where the pore size is reduced along with the depth of the pore.

**Fig. 3:**
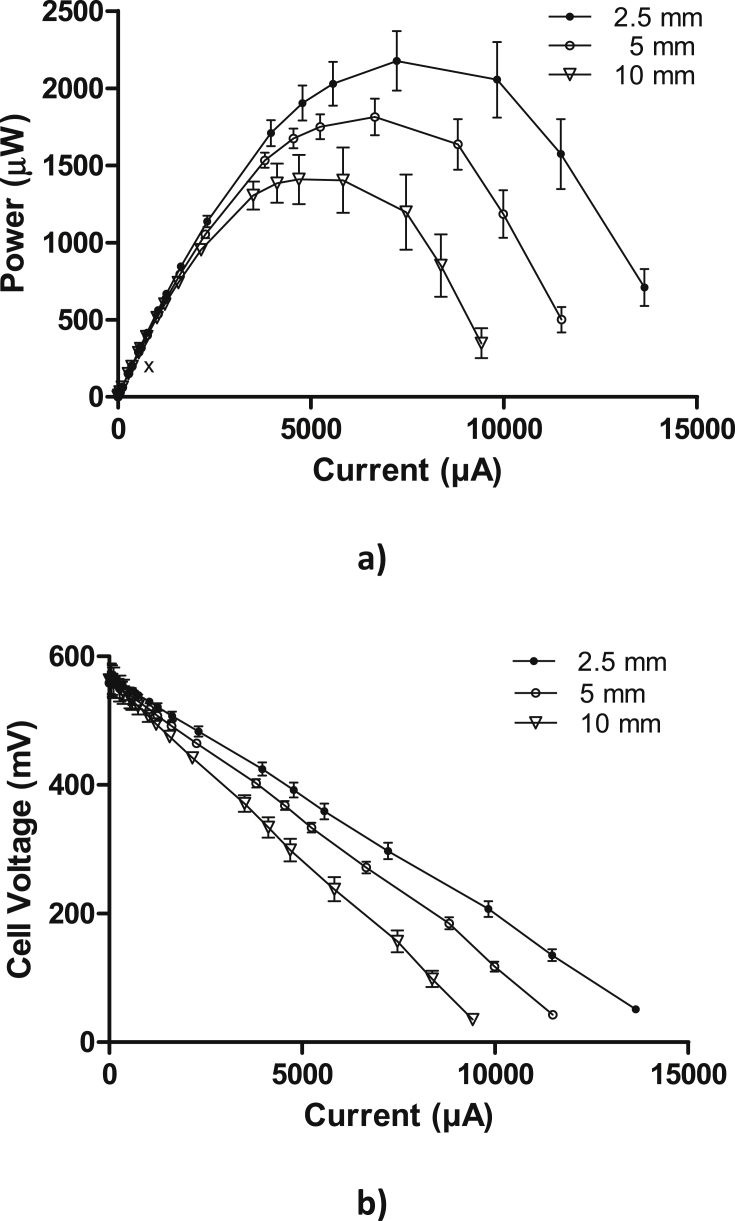
Polarisation curves obtained after 4 weeks of operation: a) Power and b) cell voltage versus current generated in the 3 different types of MFCs: MFC 1 2.5 mm FFC ceramic membrane, MFC 2 5 mm FFC ceramic membrane and MFC 3 10 mm FFC ceramic membrane. Error bars indicate SEM with n = 3.

Fig. 3 shows the comparison from the polarisation experiments performed from the three FFC thicknesses; 2.5 mm, 5 mm and 10 mm, after 4 weeks of operation. Fig. 3a shows the power produced from each MFC versus current and Fig. 3b represents the cell voltage versus current obtained during the polarisation experiment. As can be seen in Fig. 3a, the maximum power of 2.1 ± 0.19 mW was produced by the 2.5 mm FFC ceramic MFC, followed by the 5 mm FFC ceramic producing 1.8 ± 0.12 mW, whereas the 10 mm generated 1.4 ± 0.21 mW. These results suggest that in general, FFC acts as a good membrane material for MFCs fed with urine. The results also show that higher power is produced from the thinner ceramic materials. The average OCV measured before the polarisation experiments was quite consistent, for all three thicknesses, with negligible variation (∼1%); 563 mV for the thinnest, 567 mV for the medium and 568.5 mV for the thickest. As shown in Fig. 3b, the ohmic losses also changed with the thickness of the ceramic, with the highest values recorded from the thickest MFCs, and the lowest from the thinnest. The voltage drop variance between the FFC MFCs was probably due to the difference in the internal resistance of the ceramic material. To corroborate this, the internal resistance was calculated for each type of MFC using Eq. (4) obtaining values of 60.85 Ω, 75 Ω and 89.5 Ω, for the 2.5, 5 and 10 mm MFCs, respectively.

The anode and cathode voltages separately contribute to the overall MFC losses and therefore the half-cell electrode voltages and their differences with the ceramic thickness were also analysed.

**Fig. 4:**
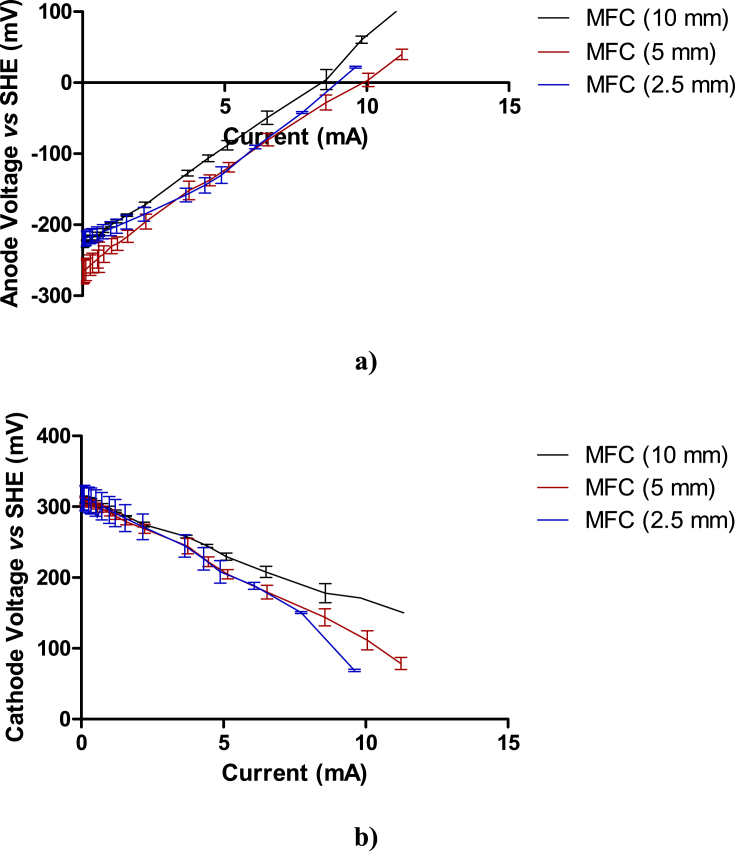
Anode voltage a) and cathode voltage b) versus SHE reference electrode as a function of the current generated in the MFCs.

Fig. 4a and b shows the anode and cathode polarisation curves, *V*_*Anode*_ and *V*_*Cathode*_, respectively obtained from one MFC of each type.

As can be seen in Fig. 4a, the activation losses were similar for all the MFCs, having the same slope in the first section of the curve. However, as previously mentioned, the data show different ohmic losses for each type of MFC. This can also be observed in the voltage drop of the MFC (10 mm, 344 mV), whereas for the 5 mm MFC and 2.5 mm MFC the drop in voltage was 323.46 mV and 259.23 mV, respectively.

Fig. 4b shows that there are also differences in the losses during the cathode polarisation between the different MFCs. The cathode open circuit voltage was approximately the same for all the MFCs, 300 mV vs. SHE, indicating no effect from the wall thickness of the ceramic membrane, on the OCV. The cathode OCV is in agreement with previously reported values for AC based cathodes [19]. The cathode voltage at zero current (0.3 V vs. SHE) was lower than the theoretical value of the ORR through a 4 electron pathway in alkaline solutions as shown in reaction (1) (0.4 V vs. SHE), suggesting a mixed reaction, involving the reduction of oxygen through the hydrogen peroxide pathway. The voltage shifting to less positive values suggests a higher contribution of the hydrogen peroxide formation, possibly leading to a less than 4 electrons reaction [11].

However, slight differences in the ohmic losses between the different types of MFC were observed, mainly for the MFC (10 mm), leading to a cathode voltage drop of 150 mV, 200 mV and 227 mV for the MFC (10 mm), MFC (5 mm) and MFC (2.5 mm), respectively. This suggests that a more favourable ORR was taking place in the MFC (10 mm), followed by the MFC (5 mm) and the MFC (2.5 mm), respectively, due to a faster oxygen reduction reaction taking place in a more alkaline media. However, the overall MFC (10 mm) power performance was limited by the anode half-cell and the higher ohmic losses from a more resistive membrane, compared to MFCs (5 and 2.5 mm). There are two factors that might cause the variation in the cathode voltage losses between the cathode polarisation curves for the different MFCs. Firstly, the wall thickness might affect the cation rate of transfer, having greater limitations to the cation flux, increasing the ORR over-potential and decreasing the cathode OCV [7]. Secondly, the differences in the catholyte accumulated in the cathodic chamber most likely have an effect on the MFC power production, by changing the pH and conductivity, and therefore the ORR voltage in the cathode [18], in addition to the ‘standard’ redox voltage. The cathode voltage is a function of the electrolyte pH, according to the Nernst equation, and it would be expected that at the maximum MFC power production, the cathode voltage for the MFCs with different thicknesses will vary with the catholyte pH.

In this case, the catholyte accumulation is a consequence of a number of factors: 1) the hydrogen peroxide produced during the ORR occurring in the cathode electrode; 2) the hydraulic pressure and fluid transport due to the MFC design, where the urine was surrounding the ceramic cylinder; 3) the concentration difference between the fluids in the anode and the cathode compartments separated by a porous ceramic material, will cause osmotic diffusion across the membrane; and 4) the electro-osmotic drag produced when the MFC is generating current, where the cations that migrate from the anode to the cathode drag water molecules along into the cathode compartment.

**Fig. 5:**
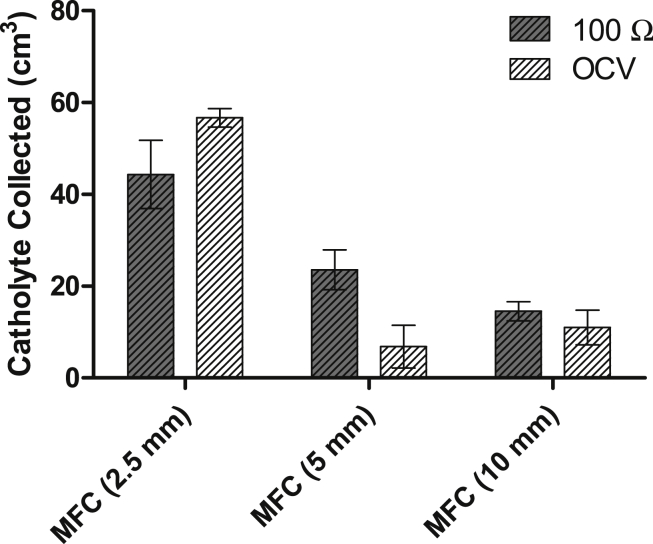
Comparison of the catholyte volume generated in 7 days of operation for the different types of MFC and the catholyte generated at the OCV.

Fig. 5 shows the amount of catholyte produced in each MFC type at the OCV and under a 100 Ω load. MFC (2.5 mm) FFC ceramic membrane produced on average 44.3 cm^3^ during 7 days of operation, which is more than 2-fold the catholyte produced in the MFCs with the 5 mm membrane (23.5 cm^3^) and more than 3-fold the catholyte produced in MFCs with the thickest membrane (14.5 cm^3^). The amount of catholyte collected from the MFCs that operated under a load of 100 Ω was significantly different to that collected from the control MFCs, under OCV conditions. The thinnest MFC produced 56.6 cm^3^ of catholyte under OCV, which is over 20% more than that generated from the MFCs (2.5 mm) under load. In contrast, the catholyte collected from the MFCs with the thicker membranes, 5 and 10 mm, produced 6.8 cm^3^ and 6 cm^3^, respectively from their control OCV MFCs, which is 71% and 58%, lower compared to the loaded MFCs respectively.

**Fig. 6:**
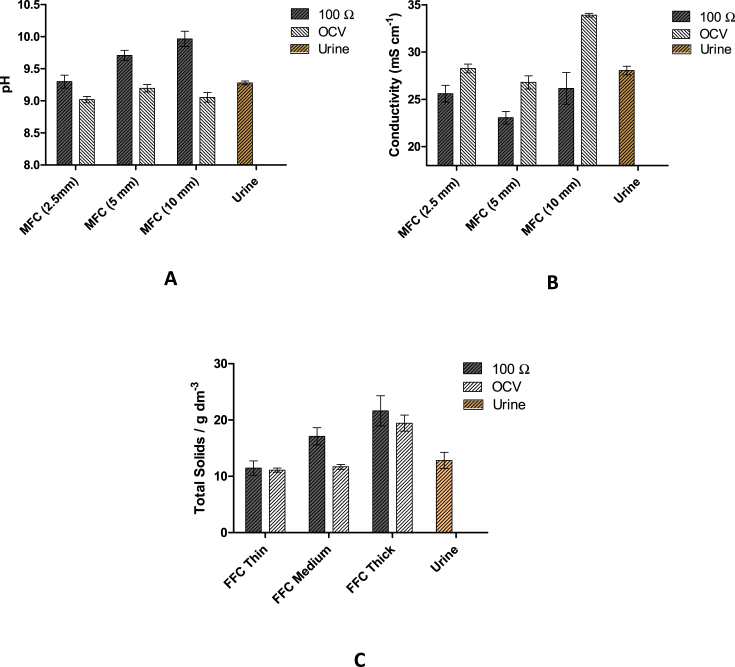
Measurements of the chemical analysis for the catholyte collected from the MFCs with different thickness under load (100 Ω) and under open circuit (OCV), in comparison with those measured from the urine used as the feedstock: A) pH, B) conductivity, C) dry weight.

Under load, a charge-balance phenomenon, electro-osmosis, is driving an ion flux from the anolyte to the catholyte. Each ion will be accompanied by a different number of water molecules, depending on the ion size and its electro-osmotic drag coefficient. It has been reported in the literature that for each K^+^ and Na^+^ cation that passes through the membrane, 11 and 3.7–6.4 water molecules will also pass through, respectively [20], [21], [22]. This helps justify the volume of catholyte generated for each thickness, when the MFCs were running under load. Synthesising more catholyte under load than under open circuit is expected, since more reactions are contributing to catholyte synthesis, ORR and electro-osmotic drag; however the paradoxically higher catholyte volume collected from the thinnest (2.5 mm) MFCs could be explained by dominant hydraulic pressure and diffusion reactions, being more pronounced compared to the thicker materials. For a thinner material, the fluid transport from the anode chamber (around the ceramic cylinder) to the initially empty cathode chamber (inside the ceramic cylinder), will be a more dominant factor contributing to the catholyte formation than in a thicker material, which poses a higher resistance to the fluid transport. These differences in catholyte generation lead to a variation in the composition, which can be evidenced by the differences in pH, conductivity and dry weight values between the different MFCs, as can be seen in Fig. 6A, B and C, respectively. The pH of the catholyte collected from the loaded MFCs increased from 9.3 to 9.71 and 10, when the wall thickness increased from 2.5 to 5 mm and to 10 mm, respectively; whereas the pH of the catholyte at OCV did not show considerable changes, across all thicknesses, remaining approximately at 9. These values are slightly lower than those of the inlet (urine), which was on average 9.25. In the thin MFCs (2.5 mm), no considerable difference between catholyte from open and closed circuit MFCs was observed. The conductivity of the thin (2.5 mm) and the medium (5 mm) MFCs at OCV was approximately the same as that of urine, except for the MFC (10 mm), whose conductivity was more than 20% higher, probably due to a more concentrated solution in a smaller liquid volume. Under load, MFCs (2.5 mm) and (5 mm), showed a decrease in the conductivity of the catholyte of 7% and 16%, respectively compared to that under OCV. This suggests the variation of the catholyte composition with the electricity production is probably due to the electro-osmotic drag.

The total solids accumulation increased with increasing wall thickness of the ceramic membrane, containing an average of 20 g dm^−3^ of total solids in the catholyte from the thickest membrane MFC, compared to 12.8 g dm^−3^ measure from neat urine. In general, the concentration of salts in the accumulated catholyte is higher than that of urine.

Table 1 shows the concentration of cations and anions present in the catholyte collected from the FFC MFCs with different thicknesses. The concentration of cations, such as Na^+^, K^+^ and Ca^2+^ increased with the thickness of the ceramic. On the contrary, the concentration of anions, including Cl^−^, ${{\text{PO}}_{4}}^{3-}$ and ${{\text{SO}}_{4}}^{-}$ in the catholyte decreased with the thickness of the ceramic membrane, when the MFCs where under load. Under OCV the concentration of anions was similar to that of the urine, which was expected since the catholyte accumulation in this case is driven only by passive diffusion and hydraulic and osmotic pressure. The rich concentration of potassium and phosphate indicates the possibility of nutrient recovery from urine for fertiliser purposes. Moreover, the ion present at the highest concentration was ammonium, which could potentially be volatised into ammonia (NH_3_) at a high pH solution [23]. Ammonia could then be recovered from the gas stream leaving the cathode. This would be yet another advantage of the MFC technology, adding NH_3_ stripping to this innovative concept of urine treatment, catholyte production and electricity generation [24].

As can be seen, the amount of catholyte and its composition changed with the ceramic wall thickness, demonstrating the influence of the hydraulic pressure and fluid transport due to the MFC design used. Variations were also observed when the system operated under open and closed circuit conditions, showing the influence of the electro-osmotic drag. The difference in pH, conductivity and dry solids between the open and closed circuit MFCs became more obvious when the wall thickness increased, since a thicker membrane prevents the flow of ions, and therefore the number of water molecules that can pass through. The more ions and water molecules passing across the membrane, the more diluted the OH^−^ produced from the oxygen reduction reaction and the lower the pH becomes.

## Conclusions

This study compares the use of fine fire clay ceramics with different thicknesses (2.5 mm, 5 mm and 10 mm) as low cost membrane materials for MFCs, for electricity generation and catholyte synthesis. The results show that the power produced decreased with increasing wall thickness, obtaining the maximum power generation of 2.1 mW per MFC from the MFCs with a membrane thickness of 2.5 mm. For the first time, the production of catholyte directly from urine, which could be used for practical applications, is presented. A further understanding on how the catholyte is generated and what parameters affect the catholyte generation and its composition is still needed. This work provides experimental evidence on the variation of the catholyte production and its composition being dependent on the membrane thickness. The amount of catholyte collected decreased with the thickness of the ceramic, while the pH and total solids increased. The catholyte generated also varied when the MFCs were operated under open and closed circuit, showing the influence of the electro-osmotic drag phenomenon. The catholyte formation and its pH also play an important role in the oxygen reduction reaction and thus it has an effect on the MFC power performance. After seven days of operation, 15 cm^3^ of catholyte at pH 10 were collected from the MFC with the thickest ceramic membrane, compared to 25 cm^3^ at pH 9.3 obtained from the thinnest MFCs. This suggests the possibility of collecting different compositions of catholyte from urine, only by changing the thickness of the ceramic membrane. Further work needs to be carried out in order to assess the effect of other parameters affecting the catholyte generation: i.e. composition changes with operation time, and the potential of water reuse from urine. Having a complete understanding of how the catholyte is generated and the parameters affecting its composition, leads to the possibility of fine tuning these parameters to obtain a high quality catholyte.

## Figures and Tables

**Table 1:** Concentration of cations and anions measured in the catholyte obtained from the different FFC MFCs.

Concentration g/l	Na^+^	${{\text{NH}}_{4}}^{+}$	K^+^	Ca^2+^	Mg^2+^	Cl^−^	${{\text{PO}}_{4}}^{3-}$	${{\text{SO}}_{4}}^{-}$
FFC Thin	1.7 ± 0.07	4.2 ± 0.4	2.1 ± 0.1	0.015 ± 0.004	0.0077 ± 0.005	1 ± 0.03	1.01 ± 0.06	1.13 ± 0.08
FFC Thin OCV	1.9 ± 0.07	5.0 ± 0.3	1.9 ± 0.2	0.013	0.003	1.21	1.12	1.35
FFC Medium	2.34 ± 0.3	4.6 ± 0.2	3.0 ± 0.3	0.015 ± 0.004	0.003	0.8 ± 0.04	0.85 ± 0.08	0.88 ± 0.02
FFC Medium OCV	2 ± 0.08	3.5 ± 0.2	2.4 ± 0.2	0.016	0.003	0.95	1.0	1.1
FFC Thick	2.1 ± 0.15	4.2 ± 0.18	2.8 ± 0.2	0.025 ± 0.0004	0.007	0.64 ± 0.05	0.78 ± 0.01	0.81 ± 0.01
FFC Thick OCV	2.4 ± 0.1	5.1 ± 0.3	2.8 ± 0.2	0.036 ± 0.001	0.009			
Urine	1.9 ± 0.005	5.6 ± 0.04	2.9 ± 0.18	0.09 ± 0.004	0.046 ± 0.02	0.99 ± 0.02	0.73 ± 0.02	0.86 ± 0.02
